# Patient Specific Seizure Prediction System Using Hilbert Spectrum and Bayesian Networks Classifiers

**DOI:** 10.1155/2014/572082

**Published:** 2014-08-27

**Authors:** Nilufer Ozdemir, Esen Yildirim

**Affiliations:** ^1^Iskenderun Vocational School, Mustafa Kemal University, 31200 Iskenderun, Hatay, Turkey; ^2^Department of Computer Engineering, Mustafa Kemal University, 31200 Iskenderun, Hatay, Turkey

## Abstract

The aim of this paper is to develop an automated system for epileptic seizure prediction from intracranial EEG signals based on Hilbert-Huang transform (HHT) and Bayesian classifiers. Proposed system includes decomposition of the signals into intrinsic mode functions for obtaining features and use of Bayesian networks with correlation based feature selection for binary classification of preictal and interictal recordings. The system was trained and tested on Freiburg EEG database. 58 hours of preictal data, 40-minute data blocks prior to each of 87 seizures collected from 21 patients, and 503.1 hours of interictal data were examined resulting in 96.55% sensitivity with 0.21 false alarms per hour, 13.896% average proportion of time spent in warning, and 33.21 minutes of average detection latency using 30-second EEG segments with 50% overlap and a simple postprocessing technique resulting in a decision (a seizure is expected/not expected) every 5 minutes. High sensitivity and low false positive rate with reasonable detection latency show that HHT based features are acceptable for patient specific seizure prediction from intracranial EEG data. Time spent for testing an EEG segment was 4.1451 seconds on average, which makes the system viable for use in real-time seizure control systems.

## 1. Introduction

Epilepsy is a neurological disorder that affects about 50 million people, in a wide age range from neonatal to elderly, around the world [1]. It is characterized by spontaneous recurrent seizures. A seizure occurs as a result of an excessive electrical discharge in a group of brain cells. Although it lasts for a short duration, a few seconds or minutes and rarely longer, a seizure is known to be handicapping for an epilepsy patient due to its unpredictable nature. However, patients with medically controllable epilepsy might sustain a normal life if they were able to predict an upcoming seizure in a reasonable time period. A recent report released by the World Health Organization shows that 70% of epilepsy patients respond to medication and can completely take their seizures under control [2]. Therefore prediction of an upcoming seizure could improve the life quality of the majority of epileptic patients by providing the time to take necessary precautions to prevent or decrease the possible severe effects of the seizure.

EEG is a widely accepted method for diagnosis of epilepsy. Recently, a number of studies have shown that EEG recordings also carry important information minutes prior to the seizure onset to distinguish between preictal and interictal states [3–15]. Besides, technological advances in digital EEG and implantable devices for seizure control [16, 17] have increased the attention to algorithms for automated epileptic seizure prediction systems based on EEG data with high sensitivity and specificity [4, 9, 18]. Most of the studies use analysis techniques for feature extraction and machine learning algorithms to classify the features extracted from EEG segments into interictal or preictal states. Various feature extraction methods are used, such as spectral power [9], wavelet coherence [19], wavelet energy and entropy [13], short-time Fourier transform [20], mean phase coherence [10], and empirical mode decomposition (EMD) [21–23]. Neural networks [20, 24] and support vector machines [9, 21, 25] are mostly preferred machine learning algorithms to classify the features extracted from EEG segments. Recently, Bayesian based methods are also employed in seizure prediction systems [26, 27].

EEG signals are spontaneous electrical brain activities that exhibit dynamic, stochastic, nonlinear, nonstationary, and also complex behaviour. Traditional data analysis techniques assume the time series to be linear and/or stationary. A relatively new signal processing technique, known as Hilbert-Huang transform (HHT), is proposed by Huang et al. in 1998 [28], specifically for analysing data from nonlinear and nonstationary processes. HHT was shown to be a powerful tool to examine biosignals, such as EEG and ECG [29]. Moreover, a recent study was conducted on a method for implementation of EMD for computationally efficient and accurate real-time analysis and the method's efficiency was shown for EEG and ECG datasets [30].

In this paper, we present a patient specific algorithm for seizure prediction based on HHT for feature extraction and Bayes networks for classification.

## 2. Materials and Methods

**Figure 1:**
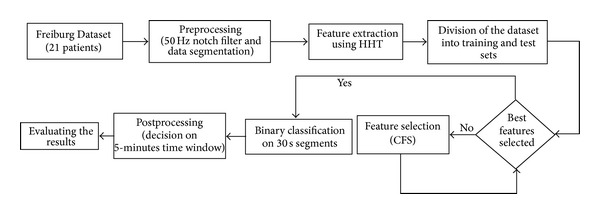
Flow chart of the proposed seizure prediction system.

In this paper we propose a method that includes preprocessing of intracranial EEG (iEEG) data for noise removal and obtaining data segments for sliding window analysis, feature extraction using Hilbert-Huang transform, feature selection using correlation based feature selection algorithm, binary classification by Bayesian networks, and a simple postprocessing algorithm to remove spurious detections. Outline of the system is given in Figure 1. System is trained and tested on the Freiburg database. Test datasets are not used in classifier modelling.

### 2.1. EEG Database

**Figure 2:**
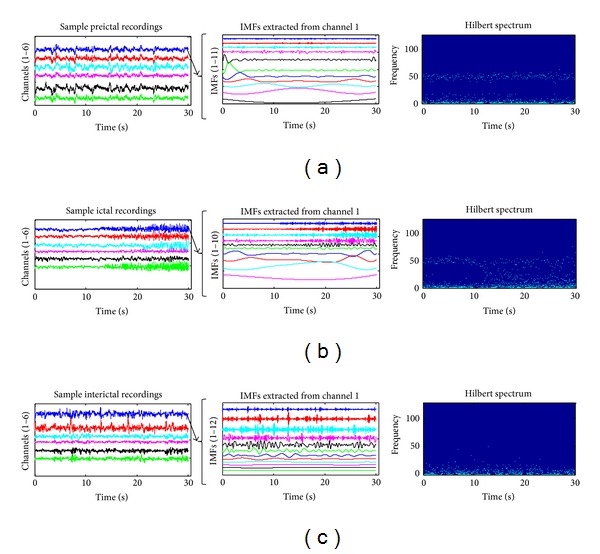
Sample EEG recordings, extracted IMFs and Hilbert spectrums for preictal activity (a), ictal activity (b), and interictal activity (c).

The Freiburg EEG database, which is available online (https://epilepsy.uni-freiburg.de/freiburg-seizure-prediction-project/eeg-database) by request, is used for training and testing the proposed system. This database contains iEEG recordings collected from 21 patients with medically intractable focal epilepsy during invasive presurgical epilepsy monitoring at the Epilepsy Center of the University Hospital of Freiburg, Germany. Recordings were acquired at 256 Hz (at 512 Hz for interictal recordings of patient 12) sampling rate with 16 bit A/D converter. Among the strip and grid electrodes used for recordings, six electrodes were identified by a certified epileptologist by visual inspection. First three electrodes (focal electrodes) are selected close to the region where the seizure occurs or the region where early ictal activity is detected, and the other three (extrafocal electrodes) are selected from the regions distal to the seizure focus or the regions in which an ictal activity is not observed. A total of 87 seizures, 504 h of interictal, and 73 h of preictal or ictal data are available in the database. At least 50 minutes of preictal data for each seizure and approximately 24 hours of iEEG data for each patient without seizure activity are available. Broader information can be obtained from [31]. Sample recordings of preictal, ictal, and interictal activities, extracted IMFs, and corresponding Hilbert spectrums for patient 2 are shown in Figure 2.

### 2.2. Preprocessing: Artifact Removal and Segmentation

We started with removing the artifacts in iEEG data, which were detected by an epileptologist visually and provided in the database in a text file. Then we filtered the data by a 50 Hz notch filter to remove the power line noise. For the analysis, we preferred moving window analysis technique and segmented the multichannel iEEG data into 30- second-long (7680 data points) windows with 50% overlap. For each window, we extracted HHT based features to feed the classifier to make a decision.

### 2.3. Hilbert Huang Transform

HHT is the designated name for an empirical and adaptive method which combines EMD, introduced by Huang et al. in 1996 [32], and very well known Hilbert spectral analysis (HSA). HHT is a time-frequency analysis technique in which the basis functions are data driven, in contrast to conventional data analysis techniques, such as Fourier transform and wavelet transform, which represent the signal by predetermined basis functions.

In this method, frequency component for each time point, called instantaneous frequency, is considered. Instantaneous frequency (IF) provides important information about the frequency content of the nonstationary signals. IF is easily obtained for complex valued signals by differentiating the phase of the signal with respect to time. However, it is ambiguous for real valued signals. A possible way to compute IF for a real valued signal, *g*(*t*), is to define an analytic signal, $\tilde{g}(t)$, by determining the complex conjugate of the signal, $\hat{g}(t)$, using the Hilbert transform [33]. Hilbert transform is simply defined as the convolution between the signal and the Hilbert transformer, 1/(*πt*). Instantaneous frequency, *w*(*t*), is defined as the derivative of the phase function with respect to time; *w*(*t*) = *dθ*(*t*)/*dt*, where *G*(*t*) is the instantaneous amplitude and *θ*(*t*) is the phase function of the analytical signal. Although the problem of obtaining the phase function is solved by the use of Hilbert transform, application of the Hilbert transform is limited to monocomponent signals [34], in which there is only one frequency component or a narrow range of frequencies varying as a function of time. After the introduction of EMD, which is used to break down a multicomponent signal into its components called intrinsic mode functions (IMF), benefits of the method became applicable for multicomponent signals. EMD is based on the local characteristics of the data instead of predefined basis functions. Therefore it is highly efficient and suitable for analysing nonlinear and nonstationary signals. Process of decomposing a signal into its IMFs is pretty well defined in the literature [28, 32].

Assuming that we have a real valued signal, *x*(*t*), EMD process is applied to the signal to decompose the signal into its IMFs. Considering the signal is decomposed into *N* IMFs, the original signal can be expressed as the sum of IMFs, *g*_*i*_(*t*), *i* = 1,…, *N*, and a residue, *r*_*N*_. Consider

(1) $$\begin{array}{c} x\left(t\right)=\overset{N}{\underset{i=1}{\sum }}g_{i}\left(t\right)+r_{N}. \end{array}$$

After obtaining IMFs, Hilbert transform is applied to extracted IMFs in order to obtain the time-frequency-energy distribution of the signal. After removing the residue, the original signal is now expressed as

(2) x(t)=Re{∑i=1NGi(t)ej∫wi(t)dt}.

Equation (2) enables us to observe the signal's energy distribution as a function of time and frequency in a 3D plot which is designated as the Hilbert spectrum, that is, *H*(*w*, *t*). Sample iEEG segments, IMFs extracted from these segments and their Hilbert spectrums are shown in Figure 2.

### 2.4. Feature Extraction

30-second-long iEEG segments for all six channels are decomposed into IMFs via EMD separately. Employing all the extracted IMFs, Hilbert spectrums of the signals are obtained. Then, the total energy, *E*_Tot_, energy in *j*th frequency band, namely, *E*_*j*_, where *j* = {*δ*(0.5–3 Hz), *θ* (4–7 Hz), *α*1 (8-9 Hz), *α*2 (10–12 Hz), *β*1 (13–17 Hz), *β*2 (18–30 Hz), *γ* (31–40, 41–50, 51–90, and 91–128 Hz)}, and contribution of *E*_*j*_ to the total energy, that is, *E*_*j*_/*E*_Tot_, are computed. Note that wide gamma band was split into four bands.

Assuming that *g*_*i*_^*k*^(*t*) is the *i*th IMF for the *k*th channel, *i* = 1,2,…, *N*, *k* = 1,2,…, 6, *H*_*i*_^*k*^(*w*, *t*) is the associated Hilbert spectrum and *H* ^*k*^(*w*, *t*) is the Hilbert spectrum of the EEG data from *k*th channel. *E*_*j*_^*k*^ is the energy in *j*th frequency band and *E*_Tot_^*k*^ is the marginal energy for the *k*th channel. Besides, groupiness factors of the energy in each frequency band are also used as features. Groupiness factor is frequently used in oceanography studies to examine wave grouping characteristics. Funke and Mansard [35] defined groupiness factor of a time series (or its spectral density) to describe the degree of grouping activity as the standard deviation of the time series over the mean of the time series. Groupiness factor based on instantaneous energy obtained by HHT was defined in [36]. Following this approach, in this study, we used groupiness factor values, standard deviation of the energy in previously defined frequency bands over the mean of the same energy values, as features. Energy in *j*th frequency band from channel *k* was used to compute *GF*_*j*_^*k*^. Listed features are estimated as in (3). Consequently, 31 features are obtained for each channel. Combining these features for each segment, finally we obtain 186 features for each window. Consider

(3) $$\begin{array}{c} H^{k}\left(w,t\right)=\overset{N}{\underset{i=1}{\sum }}H_{i}^{k}\left(w,t\right),\\ E_{j}^{k}\left(t\right)=\int_{w\in j}{\left(H^{k}\left(w,t\right)\right)}^{2}dw,\\ E_{j}^{k}=\int_{t}E_{j}^{k}\left(t\right)dt,\\ E_{\text{Tot}}^{k}=\int_{t}\int_{w}{\left(H^{k}\left(w,t\right)\right)}^{2}dw\;dt,\\ GF_{j}^{k}=\frac{\sigma_{j}^{k}}{{\bar{E}}_{j}^{k}}. \end{array}$$

### 2.5. Feature Selection

Feature selection is a technique that removes irrelevant and/or redundant features from the whole feature set and selects the most relevant ones to build a model with a better performance. Filters [37] and wrappers [38] are two general evaluation strategies for feature selection.

Wrappers search through the space of possible features and evaluate each feature subset using classifier accuracy as the objective function. Filter objective functions, on the other hand, are usually simpler measures such as information gain ratio or feature-feature and feature-class correlation. Although best performance is obtained using exhaustive search through all possible feature combinations, it is unfeasible and time consuming. Therefore, suboptimal but faster search functions, such as hill-climbing, genetic, best first, and random, are usually chosen.

In this study, we employed a filtering type of feature selection, correlation based feature selection (CFS) [39] with best first search algorithm. The fundamental idea behind this method is that a good feature set consists of the features that are correlated with the class but are not correlated with each other. In our study, the number of features is reduced from 186 to 14.49 on the average (*σ* = 6.95).  Table 1 shows the most selected features that are selected more than 15 times in 87 trials by CFS.

### 2.6. Train-Test Files

**Figure 3:**
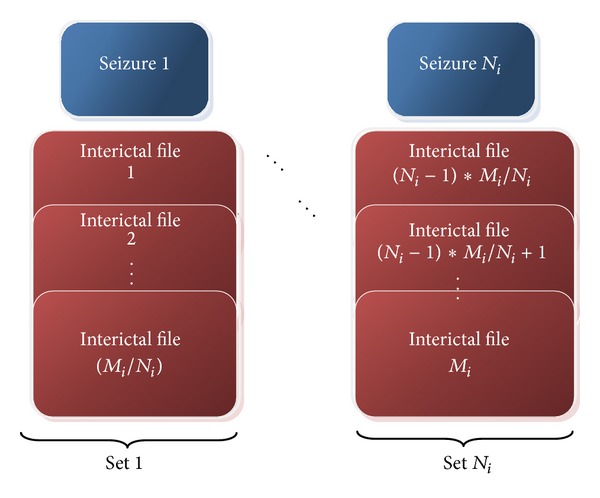
Division of dataset to be used for training and testing for *i*th patient with *N*_*i*_ seizures.

In our study we used Freiburg database for training and testing the system. Dataset used for testing is left completely out of training dataset in order to present more realistic sensitivity and specificity values. In the database there are 87 seizures for 21 patients. In order to test the system for all seizures, 87 patient specific training and testing datasets are constructed considering the number of seizures the patient has and the number of preictal files recorded for the same patient. Assuming that *i*th patient has *N*_*i*_ seizures and *M*_*i*_ files containing interictal recordings, *N*_*i*_ disjoint datasets are obtained as shown in Figure 3. In the train-test process, set *k*  (*k* = 1,2,…, *N*_*i*_) is left for testing and the remaining datasets are combined for training assuring that the test datasets are not used in classifier modelling. Therefore, contiguous recordings, which are highly correlated and might result in overoptimistic specificity results, were included either in training or in testing sets. Note that interictal files contain about 1-hour-long recordings (*μ*_filelength_ = 58.58 minutes, *σ*_filelength_ = 6.97 minutes).

### 2.7. Bayesian Networks Classifiers

Bayesian networks have become increasingly popular and were applied in various different areas, for example, biology, financial applications, causal learning, computer games, computer vision, data mining, medicine, natural language processing, and speech recognition [40]. Bayesian network method [41] is a directed acyclic graph (DAG) that represents the joint probability distribution over a set of random variables and searches for the causal connections between variables using probability and graph theory [42].

Considering a finite set of random variables, *X* = {*X*_1_,…, *X*_*n*_}, a Bayesian Network, *B*, for *X* is a directed acyclic graph. A DAG includes nodes, which correspond to the random variables, and edges which represent the conditional dependencies. Each node is annotated with a conditional probability distribution, that is, Θ, which represents *P*(*X*_*i*_∣par(*X*_*i*_)) where par(*X*_*i*_) denotes the parents of *X*_*i*_. Finally the Bayesian Network represents a unique joint probability distribution over *X*:

(4) PB(X1,…,Xn)=∏i=1nPB(Xi ∣ par(Xi))=∏i=1nΘXi ∣ par(Xi).

To use a Bayesian Network as a classifier, we define the variable set as *X* = {*X*_1_,…, *X*_*n*_, *C*}, where *X*_*i*_ are the attributes and *C* is the class variable. A Bayesian Network, *B* with joint probability distribution *P*_*B*_(*X*_1_,…, *X*_*n*_, *C*) is formed and for a given set of attributes *x*_1_,…, *x*_*n*_, the class label *c*, which maximizes the posterior probability *P*_*B*_(*c*∣*x*_1_,…, *x*_*n*_), is obtained based on the network *B*.

In this work, we used WEKA implementation of Bayesian networks [43, 44] for classification.

### 2.8. Postprocessing

**Figure 4:**
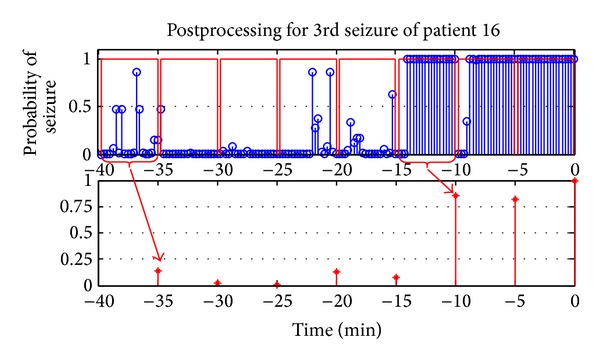
An example of postprocessing procedure shown for 3rd seizure of patient 16. Upper row shows the probability of having a seizure in near future obtained every 15 seconds (red rectangles show 5-minute windows). Lower row shows the mean probabilities of having a preictal state in 5-minute time intervals.

**Figure 5:**
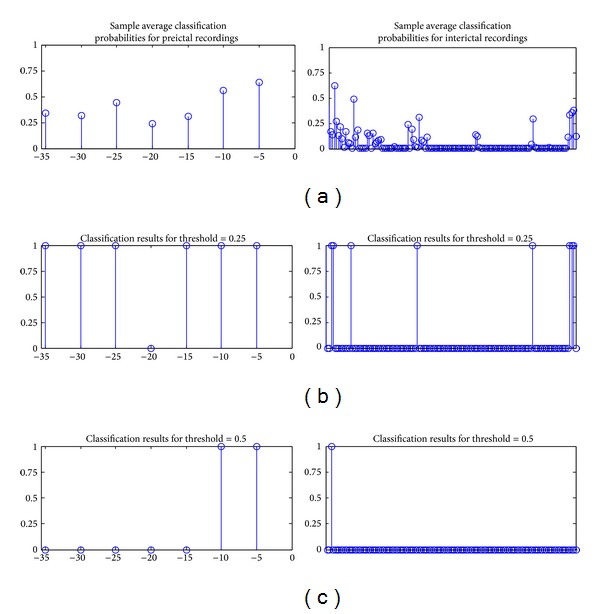
Sample demonstration of thresholding. Upper row shows the probability of having a seizure in near future. Middle row shows the classification results using 0.25 as threshold. For this preictal recordings, seizure is accepted, predicted 35 minutes prior to the seizure; that is, detection latency is 35 minutes. For the interictal recordings number of false positives is recorded as 5; consecutive alarm in a SPH + SOP window is counted as one. Lower row shows the classification results using 0.5 as threshold and the detection latency now 10 minutes and number of false positives is 1.

The ultimate goal of the study is to define a system which could predict an upcoming seizure with sufficient time to prepare for the seizure or to take the necessary precautions to prevent it. The system could be used in or with an implantable device and triggers an alarm if a seizure is expected. In presented system, such a decision is made every 15 seconds. Considering the negative effects of hearing an alarm every 15 seconds when a seizure is expected, very simple postprocessing is performed by making a decision every 5 minutes depending on the classification probabilities in this time window. The average probability of having a preictal state is computed for every 5-minute decision window. This average probability value is used for thresholding in performance assessment. For instance, for a threshold value of 0.25, a preictal classification is made only if the average probability in the five-minute decision window is higher than 0.25 and no seizure is expected in near feature (interictal classification) otherwise.  Figure 4 shows an example of the postprocessing applied on 40-minute-long preseizure data.  Figure 5 shows sample demonstration of thresholding process for preictal and interictal recordings.

## 3. Results and Discussion

We tested our patient specific algorithm for all patients in the Freiburg dataset. We examined a total of 58 hours (87 seizures ∗ 40 min/seizure) of preictal data and 503.1 hours of interictal data. Results are presented in sensitivity, mean detection latency, number of false positives per hour (FPs/h), and time spent in warning (FP%) measures to show the performance of the algorithm. Detection latency is defined as the time between the alarm and the seizure onset.

Sensitivity is calculated as the ratio of correctly predicted seizures to all seizures. A correct prediction is assessed in the sense of seizure prediction horizon (SPH) and seizure occurrence period (SOP). SOP is the time period during which the seizure onset is expected and SPH is defined as the minimum time window between the alarm and the beginning of the SOP [45]. In case of an alarm, the system holds on for a time period of SPH + SOP; if a seizure occurs in this process prediction is recorded as a true positive and as a false positive otherwise. In this study we define SPH as 5 minutes and SOP as 35 minutes. A prediction is accepted as correct only if the seizure occurs in a time period of 35 minutes starting five minutes after the alarm. Therefore if an alarm is triggered the patient will have at least five minutes to prepare but at most 40 minutes to wait for the seizure onset. Mean detection latency is recorded as the average time period between the alarms of true predictions and the following seizure onsets.

In a realistic seizure prediction system, false positives are inevitable. Number of excessive false positives, however, would decrease the credibility of the system. Therefore, an acceptable prediction system should provide a low number of false positives while resulting in high sensitivity. Number of false positives per hour (FPs/h) is a frequently used measure to evaluate the seizure prediction systems. FPs/h is computed as the division of the number of false predictions to the total interictal time examined, in hours, in system evaluation. Time spent in warning is also provided for each patient. A sample set of classification results for interictal recordings is given in Figure 7. Note that in this representation number of FPs is 2 and the time spent in warning is 120 minutes.

**Figure 6:**
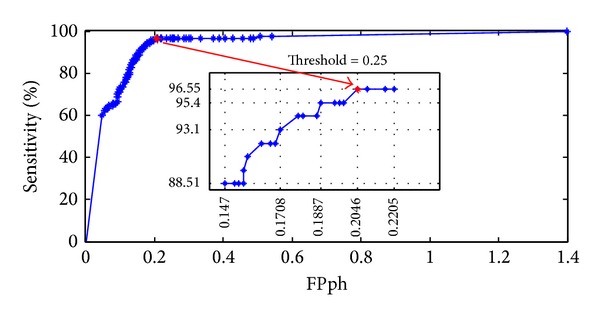
Sensitivity versus FPs/h values. Best sensitivity (96.55%) for lowest FP/h (0.2046) point is marked with a red dot.

**Figure 7:**
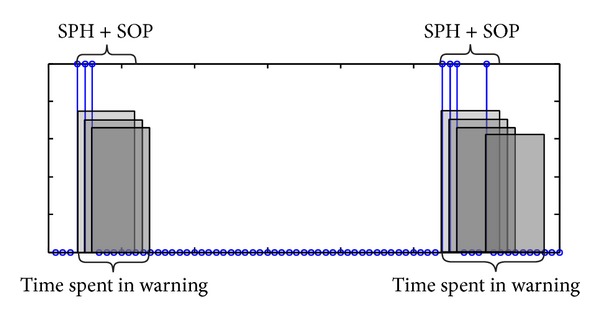
Classification results for interictal recordings of 6th patient's first test set and graphical representation for time spent in warning.

Figure 6 shows the sensitivity versus FP/h values for different threshold values. Threshold value of 0.99 in postprocessing yields 59.77% sensitivity with a FP/h rate of 0.048. Sensitivity quickly increases to 88.51% for a FP/h value of 0.147. Best sensitivity (96.55%) for the lowest FP/h (0.205) value is obtained for the threshold value of 0.25. For this point on the curve, a preictal classification is made only if the average classification probability in a five-minute time interval is higher than or equal to 0.25; that is, at least half of the windows are classified as ictal with a probability of 50% or more on the average.

Table 2 shows the seizure prediction results, corresponding to the FP/h value of 0.205, using the whole feature set and using only the features that are selected to be relevant and necessary using CFS algorithm. Note that CFS is employed independently for each trial. CFS dramatically decreased the number of features to be used for seizure prediction which shows that the feature sets chosen in the beginning are highly correlated with each other. Another result of the correlated features is that there were 11 features that are not selected in any trial. These features are listed in Table 3. Features selected more than 15 times by CFS algorithm in all trials (87 trials; one for each seizure) are shown in Table 1. Table shows that the total energy values in different frequency bands, mostly in *γ* band, are effective features in seizure prediction. However, the number of times the features used in the trials shows that there is no feature that is dominantly used in the trials. Mostly chosen feature, *E*_*γ*_4__^4^, was selected in 31 trials. Besides, features selected for each patient are noticeably different from each other and therefore starting feature selection with a large feature set is reasonable for a better chance of finding the best feature set for the patient, although the final feature set is dramatically reduced after CFS.

Results show that presented prediction system has very high sensitivity of 96.55 with a low rate of false positives, compared to the seizure prediction systems tested on the Freiburg EEG database. Earliest studies on Freiburg dataset resulted in less than 60% sensitivity [3, 31, 45], with controlled false positive rates. Average sensitivity for FP/h rate of 0.25 was reported as 57.95% in [31], and maximum %42 sensitivity was achieved for 0.15 FP/h value in [3, 45]. Later studies achieved better performances: 77.8% sensitivity for 9 patients [46], 71% sensitivity with no false positives for 15 patients [47], and 74.2% sensitivity with less than 0.2 FPs/h for all patients [19]. In [11], 11 patients with focal neocortical epilepsy were examined using a rule based prediction system and 79.9% and 90.2% of the 49 seizures were predicted for 30 min and 50 min SOP values with 0.17 and 0.11 FPs/h, respectively. For the patients with 3 or more seizures, 95% sensitivity was achieved in [14]. The best results on Freiburg database are demonstrated in [9] for the same patients with 97.5% sensitivity (78 of the 80 seizures were successfully predicted) and FPs/h rate of 0.27 for 18 of the 21 patients. In the study linear features of spectral power and support vector machines were used along with bipolar preprocessing and Kalman filters for postprocessing. In this study, we examined all patients in the database and obtained comparable sensitivity (96.55%) with better FPs/h rate of 0.21 on the average. Besides the detection latency is presented as 33.21 minutes. Note that if patients 2, 8, and 13 were excluded, as in the aforementioned study, sensitivity would be equivalent (78 out of 80) to the sensitivity reported in [9] with a better FP/h rate of 0.22 and the detection latency would be 33.46 minutes.

In order to statistically validate the results, the prediction performance of the system is compared to a random predictor. Basically, a valid prediction method should be superior to an unspecific random predictor that makes no use of any information contained in the EEG data.

### 3.1. Statistical Validation

A prediction method is considered to perform above chance level only if the performance is shown to be statistically significant. We tested our seizure prediction against a chance predictor, which is described in [8]. The chance predictor is based on a Poisson process where the interval between two consecutive alarms follows an exponential distribution [5, 8]. Note that most of the seizure prediction systems use this random predictor to show the significance of the results presented [3, 5, 8–13].

Sensitivity of the chance predictor is defined as

(5) $$\begin{array}{c} S_{nc}=1-\exp\left(-\lambda_{w}\left(\text{SPH}+\text{SOP}\right)+\left(1-e^{-\lambda_{w}\text{SPH}}\right)\right), \end{array}$$

where *λ*_*w*_ is the Poisson rate for the chance predictor which is calculated as

(6) $$\begin{array}{c} \lambda_{w}=-\frac{1}{\left(\text{SPH}+\text{SOP}\right)}\ln\left(1-\rho_{w}\right), \end{array}$$

where *ρ*_*w*_ is the proportion of time spent in warning.

To assess the significance of the system's sensitivity, *P* value of the null hypothesis should be lower than a predefined value, mostly chosen to be 0.05. Null hypothesis is stated as “*The sensitivity of the system presented is not different than the sensitivity of the chance predictor.*” Given that the algorithm under evaluation correctly predicts *n* of *N* seizures, the one sided *P* value is estimated as [8]

(7) $$\begin{array}{c} P=1-\overset{n-1}{\underset{k=0}{\sum }}\left(\begin{array}{c} N\\ k \end{array}\right)S_{nc}^{k}{\left(1-S_{nc}\right)}^{N-k},\;\text{for}\;\;\frac{n}{N}\geq S_{nc}. \end{array}$$

Prediction results are shown to be significantly better than a chance predictor for all patients except patient 19.

## 4. Conclusions

In this paper, a seizure prediction system based on Hilbert-Huang transform and Bayesian classification is presented. The system, basically, has 4 steps after preprocessing, extracting the features using HHT, selecting the best features for each patient by CFS, classification by means of Bayesian networks, and applying a simple postprocessing algorithm which combines the individual probabilities, obtained after classification task, in a 5-minute window. Results show that such a system can successfully predict the seizures with high sensitivity, 96.55% (84 out of 87 seizures) and a low false positive rate of 0.21 per hour (about one false alarm every 4.88 hours on the average). Sensitivity results are validated to be significantly better than a chance predictor for all patients except patient 19 for a significance level of 0.05. Excluding patient 19, the sensitivity would be 97.59% (81 of 83 seizures) for a FP/h rate of 0.173 (11.92%).

The system perfectly predicts the seizures with a FP/h rate of lower than 0.08 (1 FP in 12 hours) at least 31 minutes prior to the seizure onsets for nine patients (patients 1–4, 7, 9, 12, 15, and 21). Only 3 seizures (one for each patients 11, 13, and 19) were missed by the system. For four of the remaining patients, seizures were predicted at least 20 minutes before the seizure occurs with a chance of having only one false alarm in 3 hours to 5 hours.

Presented system is a patient specific seizure prediction system in which the system is trained and tested for each patient separately. By examining the features selected for each patient by CFS, there was no remarkable feature that is significant for all patients. Therefore we can conclude that the patterns followed by the extracted features for each patient are different from each other and each patient should be administered separately with the presented system. In general, however, the energy contribution of the frequency bands are shown to be the mostly selected features for the system. Note that the band that makes the contribution is different for each patient.

Performance of this system may be compared to other seizure prediction systems which used the Freiburg EEG database for training and testing. Some algorithms resulted in low sensitivity (mostly less than 50%) [3, 31, 45]. Better results are presented in other studies; however, in most of the studies, results are given only for selected patients [9, 47]. The best results on Freiburg database are demonstrated in [9] with 97.5% sensitivity with a FPs/h rate of 0.27 for 18 of the 21 patients. In this study, we examined all patients of the database and obtained comparable sensitivity (96.55%) with a better FPs/h rate of 0.21 on the average, noting that the same sensitivity is obtained for a FPs/h rate of 0.22 for those 18 patients examined in [9]. Besides detection latency is also reported as 33.21 minutes on average.

The system was trained and tested on an average desktop computer and analysis is performed using MATLAB. Time spent for processing a 30-second EEG segment was 4.1451 seconds on average, which is much smaller than required time to process 30-second data with 50% overlap (15 seconds) which makes the system viable for use in a real-time system. Average time spent on each step is as follows: 0.0754 seconds for converting database file format (.asc) to MATLAB data file format (.mat), 3.7848 seconds for obtaining IMFs from filtered data, 0.1986 seconds for extracting features for all channels and combining them into a one feature set, and 0.0863 seconds for testing and classifying the segment using the model based on the training set and applying postprocessing.

This study shows that HHT is a sensible method to extract the relevant features in a seizure prediction system. The system may be improved by using different preprocessing algorithms, choosing the optimal channels for feature extraction and employing wrapper methods for feature selection to optimize the classifier performance.

## Figures and Tables

**Table 1:** Most selected features selected by CFS.

Channel	Feature
Channel 4	Total energy in γ (91–128)
Channel 2	Total energy in γ (41–50)
Channel 4	Total energy in γ (51–90)
Channel 6	Total energy in γ (91–128)
Channel 4	Total energy in γ (41–50)
Channel 5	Total energy in γ (51–90)
Channel 6	Total energy in γ (41–50)
Channel 6	Total energy In γ (51–90)
Channel 5	Total Energy in γ (91–128)
Channel 1	Total energy in γ (41–50)
Channel 1	Total energy in γ (91–128)
Channel 2	Total energy in α1 (8-9)
Channel 3	Total energy in γ (91–128)
Channel 2	Total energy in γ (51–90)
Channel 2	GF in γ (91–128)
Channel 5	Total energy in γ (41–50)
Channel 6	Total energy in β2 (18–30)

**Table 2:** Evaluation of the proposed seizure prediction system.

Patient number	Number of Sz.	Interictal hours	CFS					No feature selection				
			Sens. (%)	Det. Lat. (min)	FPs/h	FP%	*P* value	Sens. (%)	Det. Lat. (min)	FPs/h	FP%	*P* value
1	4	23.91	100	35	0	0	0	100	27.5	0	0	0
2	3	23.89	100	35	0.08	5.178	0.0001	66.67	27.5	0	0	0
3	5	23.73	100	34	0	0	0	100	29	0.08	5.296	0
4	5	23.91	100	33	0.04	2.500	0	100	33	0.04	2.500	0
5	5	23.64	100	27	0.51	35.000	0.0031	80	23.75	0.63	40.625	0.0067
6	3	23.56	100	35	0.42	33.443	0.0272	100	33.33	0.30	21.967	0.0075
7	3	24.50	100	35	0	0	0	100	35	0.16	10.760	0.0009
8	2	24.06	100	20	0.21	17.105	0.023	100	22.5	0.21	14.803	0.0172
9	5	23.84	100	35	0	0	0	100	35	0.04	2.477	0
10	5	24.35	100	35	0.21	16.109	0.0001	100	35	0.33	27.964	0.001
11	4	23.02	75	35	0	0	0	75	35	0.22	18.301	0.015
12	4	24.63	100	35	0.04	2.462	0	100	35	0.12	9.846	0.0001
13	2	23.78	50	35	0	0	0	0	—	0	0	1
14	4	23.30	100	31.25	0.39	25.566	0.0027	75	30	0.56	41.100	0.1485
15	4	23.75	100	35	0.08	6.349	0	100	35	0.25	17.143	0.0005
16	5	23.92	100	29	0.42	24.768	0.0005	100	31	0.42	24.768	0.0005
17	5	23.98	100	35	0.29	18.154	0.0001	100	35	0.38	21.231	0.0002
18	5	24.79	100	35	0.20	13.773	0	100	35	0.40	22.455	0.0003
19	4	24.25	75	35	0.82	53.125	0.2931	75	35	0.78	53.125	0.2931
20	5	24.79	100	35	0.52	32.537	0.0021	80	35	0.65	48.358	0.1224
21	5	23.85	100	31	0.04	4.334	0	100	35	0.25	20.433	0.0002

	**87**	**503.45**	**96.55**	**33.21**	**0.21**	**13.896**	**0.000**	**90.8**	**32.53**	**0.28**	**19.347**	**0.000**

**Table 3:** Features never selected by CFS.

Channel	Feature
Channel 1	*GF*_α_1__, *E*_γ_1__/*E*_Tot_
Channel 2	*E*_γ_1__/*E*_Tot_
Channel 3	*GF*_β_1__, *GF*_β_2__
Channel 4	*E*_Tot_, *GF*_θ_, *GF*_α_1__, *E*_α_1__/*E*_Tot_
Channel 5	*GF*_α_1__, *GF*_γ_1__

## References

[1] Dua T, De Boer HM, Prilipko LL, Saxena S (2006). Epilepsy care in the world: results of an ILAE/IBE/WHO Global Campaign Against Epilepsy survey. *Epilepsia*.

[2] WHO Programmes and projects.

[3] Winterhalder M, Maiwald T, Voss HU, Aschenbrenner-Scheibe R, Timmer J, Schulze-Bonhage A (2003). The seizure prediction characteristics: A general framework to assess and compare seizure prediction methods. *Epilepsy and Behavior*.

[4] Mormann F, Kreuz T, Rieke C (2005). On the predictability of epileptic seizures. *Clinical Neurophysiology*.

[5] Schelter B, Winterhalder M, Maiwald T (2006). Testing statistical significance of multivariate time series analysis techniques for epileptic seizure prediction. *Chaos*.

[6] Sanei S, Chambers J (2007). *EEG Signal Processing*.

[7] Sackellares J (2008). Seizure prediction. *Epilepsy Currents*.

[8] Snyder DE, Echauz J, Grimes DB, Litt B (2008). The statistics of a practical seizure warning system. *Journal of Neural Engineering*.

[9] Park Y, Luo L, Parhi KK, Netoff T (2011). Seizure prediction with spectral power of EEG using cost-sensitive support vector machines. *Epilepsia*.

[10] Feldwisch-Drentrup H, Ihle M, le van Quyen M (2011). Anticipating the unobserved: prediction of subclinical seizures. *Epilepsy and Behavior*.

[11] Aarabi A, He B (2012). A rule-based seizure prediction method for focal neocortical epilepsy. *Clinical Neurophysiology*.

[12] Zandi AS, Tafreshi R, Javidan M, Dumont GA (2013). Predicting epileptic seizures in scalp EEG based on a variational bayesian gaussian mixture model of zero-crossing intervals. *IEEE Transactions on Biomedical Engineering*.

[13] Gadhoumi K, Lina J, Gotman J (2013). Seizure prediction in patients with mesial temporal lobe epilepsy using EEG measures of state similarity. *Clinical Neurophysiology*.

[14] Williamson JR, Bliss DW, Browne DW, Narayanan JT (2012). Seizure prediction using EEG spatiotemporal correlation structure. *Epilepsy and Behavior*.

[15] Joshi V, Pachori RB, Vijesh A (2014). Classification of ictal and seizure-free EEG signals using fractional linear prediction. *Biomedical Signal Processing and Control*.

[16] Stacey WC, Litt B (2008). Technology insight: neuroengineering and epilepsy—designing devices for seizure control. *Nature Clinical Practice Neurology*.

[17] Cook MJ, O'Brien TJ, Berkovic SF (2013). Prediction of seizure likelihood with a long-term, implanted seizure advisory system in patients with drug-resistant epilepsy: a first-in-man study. *The Lancet Neurology*.

[18] Gardner AB, Krieger AM, Vachtsevanos G, Litt B (2006). One-class novelty detection for seizure analysis from intracranial EEG. *Journal of Machine Learning Research*.

[19] Chiang C-Y, Chang N-F, Chen T-C, Chen H-H, Chen L-G Seizure prediction based on classification of EEG synchronization patterns with on-line retraining and post-processing scheme.

[20] Tzallas AT, Tsipouras MG, Fotiadis DI (2009). Epileptic seizure detection in EEGs using time-frequency analysis. *IEEE Transactions on Information Technology in Biomedicine*.

[21] Duman F, Özdemir N, Yildirim E Patient specific seizure prediction algorithm using Hilbert-Huang transform.

[22] Özdemir N, Yildirim E Epileptic seizure prediction based on hilbert huang transform and artificial neural networks.

[23] Tafreshi AK, Nasrabadi AM, Omidvarnia AH Empirical mode decomposition in epileptic seizure prediction.

[24] Schuyler R, White A, Staley K, Cios K (2007). Epileptic seizure detection. *IEEE Engineering in Medicine and Biology Magazine*.

[25] Gürsoy MI, Subaşi A A comparison of PCA, ICA and LDA in EEG signal classification using SVM.

[26] Santaniello S, Sherman DL, Mirski MA, Thakor NV, Sarma SV A Bayesian framework for analyzing iEEG data from a rat model of epilepsy.

[27] Jallon P A Bayesian approach for epileptic seizures detection with 3D accelerometers sensors.

[28] Huang NE, Shen Z, Long SR (1998). The empirical mode decomposition and the Hilbert spectrum for nonlinear and non-stationary time series analysis. *Proceedings of the Royal Society of London A: Mathematical, Physical and Engineering Sciences*.

[29] Eftekhar A, Vohra F, Toumazou C, Drakakis EM, Parker K Hilbert-huang transform: preliminary studies in epilepsy and cardiac arrhythmias.

[30] Eftekhar A, Toumazou C, Drakakis EM (2013). Empirical mode decomposition: real-time implementati on and applications. *Journal of Signal Processing Systems*.

[31] Aschenbrenner-Scheibe R, Maiwald T, Winterhalder M, Voss HU, Timmer J, Schulze-Bonhage A (2003). How well can epileptic seizures be predicted? an evaluation of a nonlinear method. *Brain*.

[32] Huang NE, Long SR, Shen Z (1996). The mechanism for frequency downshift in nonlinear wave evolution. *Advances in Applied Mechanics*.

[33] Saff E, AD S (1976). *Complex Analysis for Mathematics, Science and Enginering*.

[34] Cohen L (1995). *Time-frequency Analysis*.

[35] Funke E, Mansard E On the synthesis of realistic sea states.

[36] Saulnier J-B, Clément A, de O. Falcão AF, Pontes T, Prevosto M, Ricci P (2011). Wave groupiness and spectral bandwidth as relevant parameters for the performance assessment of wave energy converters. *Ocean Engineering*.

[37] John GH, Kohavi R, Pfleger K Irrelevant features and the subset selection problem.

[38] Kohavi R, John GH (1997). Wrappers for feature subset selection. *Artificial Intelligence*.

[39] Hall M (1999). *Correlation-based feature selection for machine learning [Ph.D. thesis]*.

[40] Neapolitan RE (2003). *Learning Bayesian Networks*.

[41] Pearl J (1988). *Probabilistic Reasoning in Intelligent Systems: Networks of Plausible Inference*.

[42] Friedman N, Geiger D, Goldszmidt M (1997). Bayesian network classifiers. *Machine Learning*.

[43] Hall M, Frank E, Holmes G, Pfahringer B, Reutemann P, Witten IH (2009). The weka data mining software: an update. *ACM SIGKDD Explorations Newsletter*.

[44] Bouckaert RR (2008). Bayesian network classifiers in weka for version 3-5-7.

[45] Maiwald T, Winterhalder M, Aschenbrenner-Scheibe R, Voss HU, Schulze-Bonhage A, Timmer J (2004). Comparison of three nonlinear seizure prediction methods by means of the seizure prediction characteristic. *Physica D: Nonlinear Phenomena*.

[46] Park Y, Parhi K, Netoff T Seizure prediction by spectral power in nine bands using cost-sensi tive svm.

[47] Mirowski P, Madhavan D, LeCun Y, Kuzniecky R (2009). Classification of patterns of EEG synchronization for seizure prediction. *Clinical Neurophysiology*.

